# Increasing Healthspan: Prosper and Live Long

**DOI:** 10.1016/j.ebiom.2015.11.015

**Published:** 2015-11-07

In this editorial of the first anniversary issue of *EBioMedicine*, we cannot help but reflect on aging. Tremendous biomedical successes over the last century have contributed to ever-increasing life expectancies. In its September 30, 2015 *World report on ageing and health*, the World Health Organization estimates that the global average life expectancy is now greater than 60 years. In developed nations, 20% of the population is older than 65 years. This proportion is expected to grow to 25% by 2050, the same time that the global life expectancy reaches greater than 70 years. An increase in lifespan, though, is not necessarily good news. Disease risk exponentially increases with age, with older populations being at greatest risk for cardiovascular disease, cancer, chronic respiratory illness, musculoskeletal diseases, and neurological and cognitive disorders. Already creaking health care systems may be unable to withstand the impending weight of over one billion elderly patients by 2050. The challenge now is not only how to increase lifespan, but also how to increase healthspan — the years one spends subjectively content and without burdensome medical needs.

For time immemorial humans have dreamed of living longer in good health, questing for the mythical Fountain of Youth. A recent surge in aging research has brought promise — and hype — to this quest. Basic science discoveries in model organisms that experimental manipulations can delay cellular senescence and increase longevity have positioned aging as not simply an inevitable, unalterable fact of life but as a modifiable biological process. Clinical studies following human patients longitudinally, while also carefully examining supercentenarians, have provided other key insights into the genetic and environmental factors affecting healthy aging. While these findings have redoubled the efforts of some for a magical anti-aging pill — a scientifically and ethically dubious pursuit — improving people's health in old age has considerable implications for health care systems facing a looming influx of elderly patients. So what interventions to increase healthspan are on the horizon?

A mounting body of evidence implicates metabolic systems and their eventual dysregulation in aging. It is widely recognized that the simplest ways to improve healthspan are through diet and exercise, which also tend to increase lifespan by abrogating co-morbidities associated with poor health. Among these behavioral interventions, calorie restriction and even intermittent fasting appear to increase regenerative capacity and reduce risk for age-related co-morbidities.

While lifestyle modifications may seem simple to implement for many, they may not be feasible for some, particularly those already afflicted by other maladies. A growing understanding of the biological processes underlying aging — the pathways modulating cell growth and proliferation *versus* senescence and death — has identified several pharmacological targets. Among the first were the sirtuin family of proteins, whose activation by the naturally-occurring compound, resveratrol, was found to increase healthspan in model organisms. However, trials in humans have not yielded promising results, and targeting sirtuins to increase healthspan remains scientifically controversial.

Other pharmacological targets for increasing healthspan are the mammalian target of rapamycin complexes 1 and 2 (mTORC1 and 2). Inhibition of mTOR with the US Food and Drug Administration (FDA)-approved drug, sirolimus, has been shown to increase longevity in a wide range of organisms, and is now being tested for pro-health effects in humans. Another mTOR inhibitor, everolimus was recently shown to improve immune response in elderly patients administered an influenza vaccine, offering hope for mTOR inhibitors in age-dependent medical needs. Upstream of mTORC2 is the insulin/insulin-like growth factor 1 (IGF-1) receptor pathway. In model organisms, direct IGF-1 treatment can extend lifespan, as can inhibition of the downstream modulatory Ras-Erk complex with the FDA-approved drug, trametinib. Whether these findings on mTORC2 translate to improve health in aged human patients is an area of active investigation — as is the investigation of mTORC1.

The first-line type 2 diabetes drug, metformin, suppresses glucose production in the liver by activating adenosine-monophosphate-activated protein kinase (AMPK). AMPK is an endogenous inhibitor of mTORC1, and the activation of AMPK by metformin has been shown in model systems and early clinical studies to reduce cell senescence and increase healthspan. These findings have led to the recently proposed Targeting Aging with Metformin (TAME) clinical trial where, for the first time, the indication for treatment would be aging itself. Since aging *per se* is not currently recognized as a disease, having no clear clinical endpoint, researchers began discussions with the FDA in June 2015 to hammer out specifics of the study. Aging, by far above all other factors, increases the risk for most diseases, many of which are co-morbid in the elderly. If metformin treatment is able to improve healthspan, TAME researchers argue, the age-dependent increased risk for cancer, cardiovascular disease, and cognitive decline — measurable clinical endpoints — should be dampened. Opponents argue that recognizing aging as a disease will cement prejudices against the aged. Nevertheless, the TAME study has incited policy makers to seriously consider the ramifications of aging at both the patient and health care system levels.

With recent advances in understanding the processes behind aging and the clinical needs of a growing elderly population, the focus of current translational aging research appears to be, wisely, shifting away from simply increasing lifespan to increasing healthspan. Living longer can be seen as a beneficial side effect of improving health in old age. As *EBioMedicine* ages beyond its first year, we also aspire to increase our healthspan by being the destination journal for publishing groundbreaking translational research, the side effect of which will be many more issues for years to come.

-*EBioMedicine*

